# Influence of body mass index and age on day-of-surgery discharge, prolonged admission, and 90-day readmission after fast-track unicompartmental knee arthroplasty

**DOI:** 10.1080/17453674.2021.1968727

**Published:** 2021-08-20

**Authors:** Christian Bredgaard Jensen, Anders Troelsen, Pelle Baggesgaard Petersen, Christoffer Calov JØrgensen, Henrik Kehlet, Kirill Gromov, on behalf of the Centre for Fast-Track Hip and Knee Replacement Collaborative Group

**Affiliations:** 1 Department of Orthopaedic Surgery, Clinical Orthopaedic Research Hvidovre, Copenhagen University Hospital Hvidovre, Hvidovre 2650, Denmark; 2 Section for Surgical Pathophysiology, Rigshospitalet, Copenhagen 2100, Denmark; 3 Centre for Fast-track Hip and Knee Arthroplasty, Rigshospitalet, Copenhagen 2100, Denmark

## Abstract

Background and purpose — The indications for unicompartmental knee arthroplasty (UKA) have become less restrictive and, today, high age and high BMI are not considered contraindications by many surgeons. While the influence of these patient characteristics on total knee arthroplasty is well documented, evidence on UKA is lacking. We investigated the effect of BMI and age on day of surgery (DOS) discharge, prolonged admission, and 90-day readmission following UKA surgery.

Patients and methods — This retrospective cohort study included 3,897 UKA patients operated on between 2010 and 2018 in 8 fast-track arthroplasty centers. Patients were divided into 5 BMI groups and 5 age groups. Differences between groups in the occurrence of DOS discharge, prolonged admission > 2 days, and 90-day readmission was investigated using a chi-square test and mixed-effect models adjusted for patient characteristics using surgical center as a random effect.

Results — Median LOS was 1 day. DOS discharge was achieved in 26% of patients with no statistically significant differences between BMI groups. DOS discharge was less likely in UKA patients aged > 70 years (age 71–80; odds ratio [OR] 0.7 [95% CI 0.6–0.9]). Prolonged admission was not affected by BMI or age in the adjusted analysis. 90-day readmission was more likely in patients with BMI > 35 (OR 1.9 [CI 1.1–3.1]) and patients aged 71–80 (OR 1.5 [CI 1.1–2.1]).

Interpretation — Age > 70 years decreased the likelihood of DOS discharge after UKA. High BMI as well as advanced age increased the likelihood of 90-day readmission. This should be noted by surgeons operating on patients with high BMI and age.

The indications for unicompartmental knee arthroplasty (UKA) as treatment for osteoarthritis (OA) have become less restrictive in terms of age and weight. Early contraindications included age < 60 years and weight > 82 kg (Kozinn and Scott 1989). However, recent studies report that revision rates and patient-reported outcomes are not worse in such patients (Pandit et al. 2011, van der List et al. 2016, Hamilton et al. 2017). Current indications focus solely on the pathoanatomy of the knee OA (Goodfellow et al. 1988, Hamilton et al. 2017).

Despite being informed of increased risk of certain postoperative complications in high BMI patients and young/old patients, these patients are increasingly undergoing UKA surgery as well as knee arthroplasty in general (Price et al. 2018, Henkel et al. 2019).

While length of stay (LOS) and readmissions are not the primary factors when determining indications/contraindications of arthroplasty procedures, they do affect patient satisfaction, logistics and cost-effectiveness (Reilly et al. 2005, Molloy et al. 2017).

The few studies investigating the effect of BMI and age on LOS and readmission after UKA have varying conclusions. Some studies have associated higher BMI with increased risk of prolonged admission as well as short-term complications, while others find no such association (Otero et al. 2016, Plate et al. 2017, Sephton et al. 2020). Likewise, day of surgery (DOS) discharge is reported to be less likely in older patients while some do not find this association (Haughom et al. 2015, Matsumoto et al. 2020). Due to the usage of UKA in patients with high BMI and advanced age, it is important to investigate the effect of BMI and age on the postoperative course after UKA.

We therefore investigated the association between BMI and age and the proportion of UKA patients with DOS discharge, prolonged admission, and readmission within 90 days of surgery in a prospective unselected multicenter fast-track setup.

## Patients and methods

This study was an observational cohort study investigating primary UKA procedures. All data was acquired from the Lundbeck Foundation Centre for Fast-track Hip and Knee Replacement Database (LCDB).

A previous study regarding this cohort of UKA patients from LCDB as well as multiple studies describing the data methodology within the LCDB have been published (Jørgensen and Kehlet 2013, Gromov et al. 2020). Data regarding UKA patients from the LCDB is gathered from 8 surgical centers in Denmark between 2010 and 2018. All included centers have implemented a fast-track setup with intended spinal anesthesia, multimodal opioid-sparing analgesia, high-dose preoperative corticosteroids (Kehlet and Lindberg-Larsen 2018), preoperative intravenous tranexamic acid, no drains, early mobilization with full weight-bearing, and discharge to the patient’s own home. All centers use functional discharge criteria (Husted 2012).

Data on length of stay and readmissions from the LCDB was obtained from the Danish National Patient Registry (DNPR), from which such data is considered complete (> 99%) (Schmidt et al. 2015), as well as chart-review in case of LOS > 4 days, 90-day readmission, or death. Preoperative comorbidity was evaluated using preoperative nurse-assisted patient-reported questionnaires and data from the Danish National Database of Reimbursed Prescriptions (DNDRP) (Johannesdottir et al. 2012), which provided information on use of anticoagulants, diabetic medication, antihypertensive treatment, and psychotropic treatment (Jørgensen et al. 2016). Length of stay was calculated as the number of nights spent in hospital. DOS discharge was defined as LOS = 0 days and prolonged admission as LOS > 2 days. Readmissions were defined as unplanned admissions with a LOS ≥ 1 day within 90 days surgery.

The exclusion criteria for the LCDB were age < 18, non-elective procedures, simultaneous bilateral procedures, additional major arthroplasty within 90 days, and surgery due to congenital disorders, infections, or cancer. Patients who did not answer the preoperative questionnaire were also excluded. 3,927 primary medial or lateral UKA procedures in 3,623 patients performed between 2010 and 2018 with a completed questionnaire on demographics and comorbidities were identified within the LCDB.

Patients were sorted into groups based on their BMI in accordance with the suggestions by the World Health Organization: BMI < 18.5 as underweight, BMI ≥ 18.5 and < 25 as normal weight, BMI ≥ 25 and < 30 as overweight, BMI ≥ 30 and < 35 as obese, BMI ≥35 and < 40 as very obese, BMI ≥ 40 as morbidly obese (World Health Organization n.d.). However, since only 8 patients (0.2%) were underweight (BMI < 18.5) the underweight group was included in the normal weight group (BMI < 25). Patients were also divided into 5 age groups: age < 50, age ≥ 50 and ≤ 60, age > 60 and ≤ 70, age > 70 and ≤ 80, age > 80.

### Statistics

Categorical data is displayed as n (%) with 95% confidence intervals (CI). Continuous data is reported as mean and standard deviation (SD) or median and interquartile range (IQR) for normal and non-normal distributions of data, respectively. Normality was investigated using histograms and Q–Q plots.

Differences in proportions of UKA patients within each BMI group with DOS discharge, prolonged admission, or 90-day readmission are compared using a chi-square test with BMI < 25 as reference category. Differences in proportions of UKA patients within each age group with DOS discharge, prolonged admission, or 90-day readmission are compared using a chi-square test with 61–70 years of age as reference category.

Adjusted analyses using multivariable mixed-effect models were conducted in order to determine the effect of age and BMI on DOS discharge, prolonged admission, and 90-day readmissions. The adjusted analyses included the BMI groups; age groups; sex; living situation (with others, alone, institution); use of walking aid; preoperative anemia; diabetes mellitus (insulin dependent [IDDM] or non-insulin dependent [NIDDM]); hypertension; use of potent anticoagulants; and pharmacologically treated cardiac disease, pulmonary disease, and psychiatric disorder. The surgical center was included as a random effect in the adjusted analyses to account for differences between centers (Figure 1, see Supplementary data). Only patients with complete data on the above-mentioned patient characteristics were included in the adjusted analyses. A p-value < 0.05 was considered significant.

Statistical analyses were conducted using R-studio and R v 3.6.1 (R Foundation for Statistical Computing, Vienna, Austria).

### Ethics, funding, and conflicts of interest

Approval from an ethical committee was not required as this is an observational study. Data retrieval for the LCDB was permitted by the Danish National Board of Health (3-3013-56/2/EMJO). Storing of data for the LCDB was approved by the Danish Data Protection Agency (P-2019-709).

No funding was received for this study. The authors declare no conflicts of interest directly related to this study.  

## Results

Due to missing data on BMI only 3,897 UKA procedures (99%) were included in the study, and of these 3,727 (96%) were medial UKA. Due to missing data in variables included in the adjusted analyses only 3,596 UKA procedures (92%) were included in these analyses. Differences in endpoint measures between included and excluded procedures regarding the adjusted analyses are displayed in Tables 1 and 2 (see Supplementary data).

Within the included cohort the median BMI was 28 (IQR 25–32), mean age was 66 (SD 9.4), years, and 54% were female (Table 3). The median LOS within all BMI and age groups was 1 day (age ≤ 70 and BMI < 35, IQR 0–1; age 71–80 and BMI 35–39.9, IQR 1–1; age > 80 and BMI ≥ 40, IQR 1–2).

**Table 3.** Distribution of BMI and age groups among all included patients (N = 3,897). Values are count (%) unless otherwise specified

BMI, n	3,897	
Median [IQR]		28 [25–32]
Normal: < 25	840 (22)	
Overweight: 25.0–29.9	1,677 (43)	
Obese: 30.0–34.9	996 (26)	
Very obese: 35.0–39.9	294 (7.5)	
Morbidly obese ≥ 40	90 (2.3)	
Age, n	3,897	
Mean (SD) [range]		66 (9.4) [26–97]
< 50	168 (4.3)	
50–60	901 (23)	
61–70	1,441 (37)	
71–80	1,198 (31)	
> 80	189 (4.9)	

### Day of surgery discharge

DOS discharge was achieved in 26% (n = 992) of included patients. The proportion of DOS discharge in UKA patients with normal BMI was 26% versus 22% in morbidly obese patients. Individual analyses of the proportion of DOS discharge within each BMI group showed no statistically significant difference compared with the reference group with BMI < 25 (Table 4).

**Table 4.** Unadjusted analyses of difference in DOS discharge, LOS >2 days and 90-day readmission between BMI groups

	DOS discharge		LOS > 2 days		90-day readmission	
BMI groups (n = 3,897)	n (%) [95%CI]	p-value	n (%) [95%CI]	p-value	n (%) [95%CI]	p-value
Normal: < 25	220 (26) [23–29]	Ref.	62 (7.4) [5.6–9.2]	Ref.	54 (6.4) [4.8–8.1]	Ref.
Overweight: 25.0–29.9	423 (25) [23–27]	0.6	112 (6.7) [5.5–7.9]	0.5	107 (6.4) [5.2–7.6]	1.0
Obese: 30.0–34.9	257 (26) [23–29)	0.9	80 (8.0) [6.3–9.7)	0.6	65 (6.5) [5.0–8.1)	0.9
Very obese: 35.0–39.9	72 (25) [20–29]	0.6	23 (7.8) [4.8–11]	0.8	32 (11) [7.3–14]	0.01
Morbidly obese ≥ 40	20 (22) [14–31]	0.4	15 (17) [9.0–24]	0.002	11 (12) [5.5–19]	0.04

DOS = day of surgery. Ref. = reference group. LOS = length of stay.

Statistically significant differences in proportion of DOS discharge were present between age groups, with the reference group aged 61–70 years being discharged on DOS in 28% of cases compared with 21% of cases aged 71–80 years and 7.4% of cases aged > 80 years (Table 5). In the adjusted analysis the 2 oldest age groups were also found to be less likely to be discharged on DOS compared with the reference group (age 71–80, odds ratio [OR] = 0.71 [CI 0.58–0.88]; age > 80, OR = 0.18 [CI 0.10–0.34]) (Table 6); while BMI had no effect on DOS discharge (Table 7). UKA patients aged 50–60 years were more likely to be discharged on DOS (OR = 1.3 [CI 1–1.6]).

**Table 5.** Unadjusted analyses of difference in DOS discharge, LOS >2 days and 90-day readmission between age groups

	DOS discharge		LOS > 2 days		90-day readmission	
Age groups (n = 3,897)	n (%) [95%CI]	p-value	n (%) [95%CI]	p-value	n (%) [95%CI]	p-value
< 50	47 (28) [21–35]	1.0	15 (8.9) [4.6–13]	0.3	10 (6.0) [2.4–9.5]	0.9
50–60	276 (31) [28–34]	0.2	66 (7.3) [5.6–9.0]	0.7	48 (5.3) [3.9–6.8]	0.7
61–70	406 (28) [26–31]	Ref.	100 (6.9) [5.6–8.3]	Ref.	82 (5.7) [4.5–6.9]	Ref.
71–80	249 (21) [19–23]	<0.001	89 (7.4) [5.9–8.9]	0.6	108 (9.0) [7.4–11]	0.001
> 80	14 (7.4) [3.7–11]	<0.001	22 (12) [7.1–16]	0.02	21 (11) [6.6–16]	0.004

DOS = day of surgery. Ref. = reference group. LOS = length of stay.

**Table 6.** Adjusted analysis of occurrence of DOS discharge, LOS > 2 days and 90-day readmission in age groups

Age groups (n = 3,596)	DOS discharge	LOS > 2 days	90-day readmission
	OR (95% CI)	OR (95% CI)	OR (95% CI)
< 50	0.85 (0.55–1.3)	1.2 (0.68–2.3)	1.2 (0.59–2.6)
50–60	1.3 (1.0–1.6)	1.2 (0.83–1.7)	1.0 (0.70–1.6)
61–70	Ref.	Ref.	Ref.
71–80	0.71 (0.58–0.88)	0.95 (0.69–1.3)	1.5 (1.1–2.1)
> 80	0.18 (0.10–0.34)	1.3 (0.73–2.2)	1.6 (0.89–2.8)

DOS = day of surgery. Ref. = reference group. LOS = length of stay. OR = odds-ratio.

**Table 7.** Adjusted analysis of occurrence of DOS discharge, LOS > 2 days and 90-day readmission in BMI groups

BMI groups (n = 3,596)	DOS discharge	LOS > 2 days	90-day readmission
	OR (95% CI)	OR (95% CI)	OR (95% CI)
Normal: < 25	Ref.	Ref.	Ref.
Overweight: 25.0–29.9	0.96 (0.76–1.2)	0.97 (0.69–1.4)	1.0 (0.73–1.5)
Obese: 30.0–34.9	1.0 (0.78–1.3)	1.1 (0.76–1.6)	0.99 (0.65–1.5)
Very obese: 35.0–39.9	0.91 (0.62–1.3)	1.0 (0.60–1.8)	1.9 (1.1–3.1)
Morbidly obese ≥ 40	1.1 (0.59–2.0)	1.5 (0.71–3.0)	2.1 (0.98–4.5)

DOS = day of surgery. Ref. = reference group. LOS = length of stay. OR = odds-ratio.

### Prolonged admission

Prolonged admission beyond 2 days occurred in 7.5% (n = 292) of included patients. Prolonged admission was more frequent in morbidly obese patients (17%) as well as patients aged > 80 years (12%) compared with patients with normal BMI (7.4%) and patients aged 61–70 years (6.9%), respectively (Tables 4 and 5). However, in the analysis adjusted for other patient characteristics and preoperative comorbidity there was no statistically significant difference in LOS > 2 days between BMI and age groups (Tables 6 and 7).

### Readmission within 90 days of surgery

The overall readmission rate within the cohort was 6.9% (n = 269). Readmission within 90 days of surgery occurred more frequently in patients with a BMI ≥ 35 (very obese 11%; morbidly obese 12% [p = 0.07]) compared with patients with normal BMI (6.4%) (Table 4). Readmissions within 90 days were also more frequent in patients aged > 70 years (aged 71–80 years, 9.0%; aged > 80 years, 11%) compared with patients aged 61–70 years (5.7%) (Table 5). In the adjusted analysis patients with BMI ≥ 35 (very obese, OR = 1.9 [CI 1.1 – 3.1]) (Table 7); and aged > 70 years (age 71–80, OR = 1.5 [CI 1.1–2.1]) remained more likely to be readmitted within 90 days compared with the BMI and age reference groups, respectively (Table 6). However morbid obesity (OR = 2.1 [CI 1.0–4.5], p = 0.06) and age > 80 years (OR = 1.6 [CI 0.9–2.8]) were not statistically significant factors in the adjusted analysis (Tables 6 and 7). DOS discharge in high BMI or high-age patients did not increase readmission rate compared with patients with LOS > 0 days (very obese with DOS discharge, 5.6% vs. very obese with LOS ≥ 1 day, 13%; age > 80 years with DOS discharge, 7.1% vs. age > 80 years with LOS ≥ 1 day = 13%) (Figures 2 and 3, see Supplementary data). 

## Discussion

UKA patients in our study had a normal BMI in roughly one-fifth of cases, 0.2% were underweight, almost half of the patients categorized as overweight (43%), and 2.3% were morbidly obese. Patients were mostly aged between 61 and 70 years (37%). Age > 70 years reduced the likelihood of DOS discharge. While age and BMI did not affect prolonged admission, high BMI and advanced age increased the likelihood of readmission within 90 days of surgery.

A study from the American College of Surgeons National Quality Improvement Program (ACS-NSQIP) reported that most UKA patients were obese and 9.2% were morbidly obese (Sundaram et al. 2019). Differences in national baseline demographics might explain the differences between these results and ours, as the baseline rate of obesity among adults in Denmark was 17% in 2017 compared with 42% in the Unites States of America in the same year (OECD 2019, Hales et al. 2020). A previous study investigating BMI in TKA patients from the LCDB found a somewhat similar distribution of BMI, while a larger proportion of TKA patients were very or morbidly obese (very obese, 11% vs. 7.5%; morbidly obese, 4.4% vs. 2.3%) (Husted et al. 2016). This coincides with multiple studies reporting differences in patient characteristics between UKA and TKA patients (Liddle et al. 2014, Drager et al. 2016).

We found that UKA patients > 70 years of age were less likely to be discharged on DOS compared with younger patients, while BMI did not affect DOS discharge. This was similar to a study by Matsumoto et al. (2020), who also reported advanced age to be a barrier in achieving DOS discharge and while BMI did not affect DOS discharge. In that study BMI did, however, appear to be higher in patients discharged on DOS. Likewise, a study by Plate et al. (2017) found no correlation between BMI and LOS after robotic-assisted UKA surgery.

Our study found morbid obesity and age > 80 years to be associated with prolonged hospitalization beyond 2 days after UKA in unadjusted analyses, but not in the adjusted analysis. Therefore, other patient characteristics and preoperative comorbidity beyond high BMI and age might influence the risk of prolonged admission. In contrast, other studies have reported higher BMI to be associated with prolonged admission (Haughom et al. 2015, Sephton et al. 2020). Differences in variables included in the adjusted analyses as well as the adherence to fast-track principles could potentially contribute to the above-mentioned discrepancies. In comparison, obesity and high BMI may increase LOS after TKA (Piuzzi et al. 2019, Shah et al. 2019).

Readmissions within 90 days of UKA surgery occurred more frequently in patients > 70 years of age and BMI > 35 (but not statistically significant in the morbidly obese patients and patients > 80 years of age in the adjusted analysis). However, other patient characteristics, preoperative comorbidity, and missing data in the adjusted analysis might account for readmissions in extreme BMI and age groups. Using data from ACS NSQIP from 2005–2012, Haughom et al. (2015) (n = 2,316) reported that obesity increased the risk of 30-day readmissions and complications. Using data from ACS NSQIP from 2008–2016, Sundaram et al. (2019) (n = 8,029) found overweight patients to have a lower risk of 30-day readmissions and complications compared with normal-weight patients, while morbidly obese patients had an increased risk of superficial surgical site infections. It is plausible that the difference between these studies could be explained by temporal improvements in perioperative care and surgical technique positively influencing the overall risk associated with UKA surgery from 2005 to 2016 (Gromov et al. 2020). Despite the potential increased risk of readmissions in high BMI and high-age patients, we find that DOS discharge in these patients does not increase the readmission rate. This coincides with other studies investigating safety of DOS discharge after UKA and knee arthroplasty surgery in general (Otero et al. 2016, Pollock et al. 2016).

Some studies investigating recovery and complications after surgery, as well as arthroplasty surgery, report better outcome in overweight/obese patients compared with normal-weight patients (Shaparin et al. 2016, Smith et al. 2020). This is labelled the “obesity paradox.” The findings of Sundaram et al. (2019) mentioned above also reported fewer complications in overweight patients. A study by Katakam et al. (2021) investigating LOS in patients undergoing total hip arthroplasty and TKA found that being either underweight or morbidly obese resulted in increased LOS. It is suggested that the relationship between BMI and various outcomes is not linear but rather quadratic, with slight overweight in elderly patients countering sarcopenia and malnutrition (Shaparin et al. 2016, Smith et al. 2020). However, patient selection is also proposed as the reason for these findings, as surgeons might refrain from operating on obese patients with additional comorbidity, thus creating a healthier cohort (Zhang et al. 2018).

### Strengths and limitations

Despite extensive research on LOS and readmission rates after TKA, the literature on these subjects in UKA patients is scarce. Data from the LCDB has a high quality due to the use of the DNRP as well as chart review regarding readmission and the use of prospectively collected patient questionnaires cross-referenced to the DNDRP regarding prescribed drugs, thus ensuring complete follow-up for all patients. Data is collected from 8 surgical centers and differences in perioperative practice between these centers could be present, although centers reporting to the LCDB are required to have implemented fast-track principles with a median LOS < 3 days for hip and knee arthroplasty surgery. The indications regarding UKA surgery could differ between surgical centers, and some surgeons could potentially use high BMI as a relative contraindication. Also, 304 included patients (8.5%) had more than one UKA procedure within the study period (> 90 days from first procedure). While this may limit the external validity of the study (Bryant et al. 2006), excluding these patients with multiple procedures could also introduce bias (Ravi et al. 2013). Despite having included a high number of patients compared with other cohort studies investigating UKA patients, our study is not powered to investigate underweight patients or differences in the specific complications leading to readmission.

In conclusion, UKA patients > 70 years are less likely to be discharged on DOS, while BMI did not affect DOS discharge. Advanced age and high BMI did not increase the likelihood of prolonged admission beyond 2 days but did increase likelihood of 90-day readmission. However, DOS discharge could still be considered safe in patients with advanced age and high BMI, as it did not increase the readmission rate in these patients. This data may be considered as part of the shared decision-making process and should be noted by surgeons operating on patients with high BMI and advanced age.

## Supplementary Material

Supplemental Material

## Supplementary data

Figures 1–3 and Tables 1 and 2 are available as supplementary data in the online version of this article, http://dx.doi.org/10. 1080/17453674.2021.1968727
