# Supplementary material for: Influence of body mass index and age on day-of-surgery discharge, prolonged admission, and 90-day readmission after fast-track unicompartmental knee arthroplasty
Source: Acta Orthop. 2021 Aug 20;92(6):722–7. doi: 10.1080/17453674.2021.1968727 (PMC8734435; doi:10.1080/17453674.2021.1968727)
Supplement: Supplemental Material [file IORT_A_1968727_SM3389.pdf]

Supplementary data

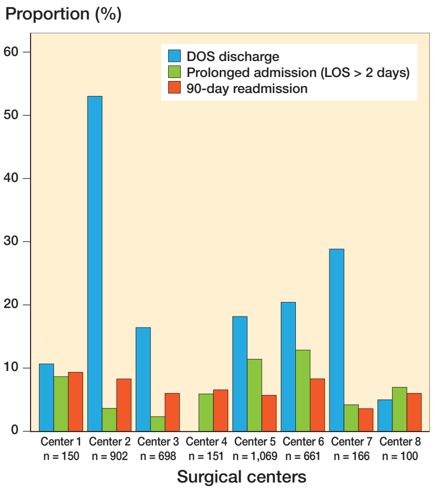

Figure 1. DOS discharge, LOS > 2 days, and 90-day readmissions sorted by surgical center.

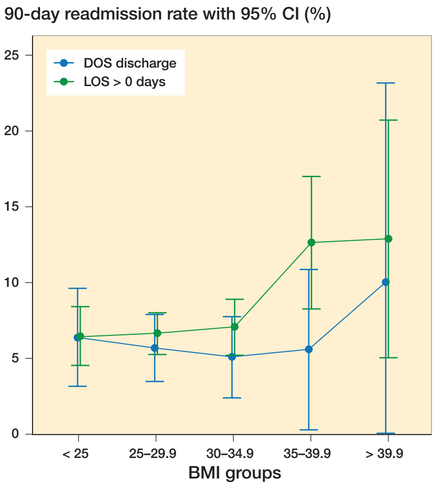

Figure 2. 90-day readmission rate (%) in patients with and without DOS discharge sorted by BMI groups.

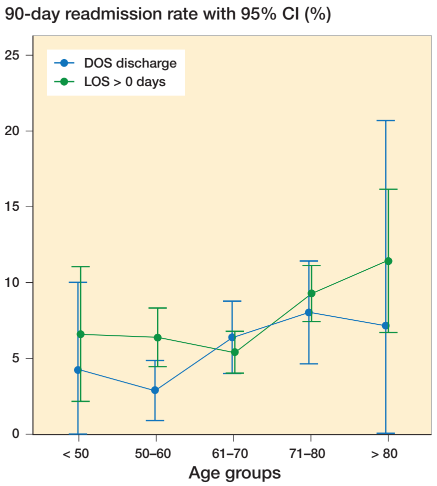

Figure 3. 90-day readmission rate (%) in patients with and without DOS discharge sorted by age groups.

Table 1. Occurrence of DOS discharge, LOS > 2 days and 90-day readmission within BMI groups sorted for patients included (n = 3,596) or not included (n = 301) in the adjusted analysis. Values are count (%)

	DOS discharge		LOS > 2 days		90-day readmission	
BMI groups	Included	Not included	Included	Not included	Included	Not included
Normal: < 25	186 (24)	34 (51)	61 (7.9)	1 (1.5)	49 (6.3)	5 (7.5)
Overweight: 25.0–29.9	361 (23)	62 (45)	111 (7.2)	1 (0.7)	102 (6.6)	5 (3.7)
Obese: 30.0–34.9	223 (24)	34 (55)	79 (8.5)	1 (1.6)	59 (6.3)	6 (9.7)
Very obese: 35.0–39.9	61 (23)	11 (39)	22 (8.3)	1 (3.6)	30 (11)	2 (7.1)
Morbidly obese ≥ 40	19 (23)	1 (14)	12 (15)	3 (43)	10 (12)	1 (14)

Table 2. Occurrence of DOS discharge, LOS > 2 days and 90-day readmission within age groups sorted for patients included (n = 3,596) or not included (n = 301) in the adjusted analysis. Values are count (%)

	DOS discharge		LOS > 2 days		90-day readmission	
Age groups	Included	Not included	Included	Not included	Included	Not included
< 50	33 (22)	14 (78)	15 (10)	0 (0.0)	9 (6.0)	1 (5.6)
50–60	226 (28)	50 (50)	63 (7.9)	3 (3.0)	44 (5.5)	4 (4.0)
61–70	341 (26)	65 (54)	97 (7.4)	3 (2.5)	74 (5.6)	8 (6.6)
71–80	237 (21)	12 (24)	88 (7.7)	1 (2.0)	104 (9.1)	4 (8.0)
> 80	13 (7.3)	1 (8.3)	22 (12)	0 (0.0)	19 (11)	2 (17)
